# Supplementary material for: Studying Plant–Insect Interactions through the Analyses of the Diversity, Composition, and Functional Inference of Their Bacteriomes
Source: Microorganisms. 2022 Dec 22;11(1):40. doi: 10.3390/microorganisms11010040 (PMC9863603; doi:10.3390/microorganisms11010040)
Supplement: Supplementary file 1 [file microorganisms-11-00040-s001.zip › microorganisms-2045956-supplementary.pdf]

SUPPLEMENTARY MATERIAL

Supplementary Figures

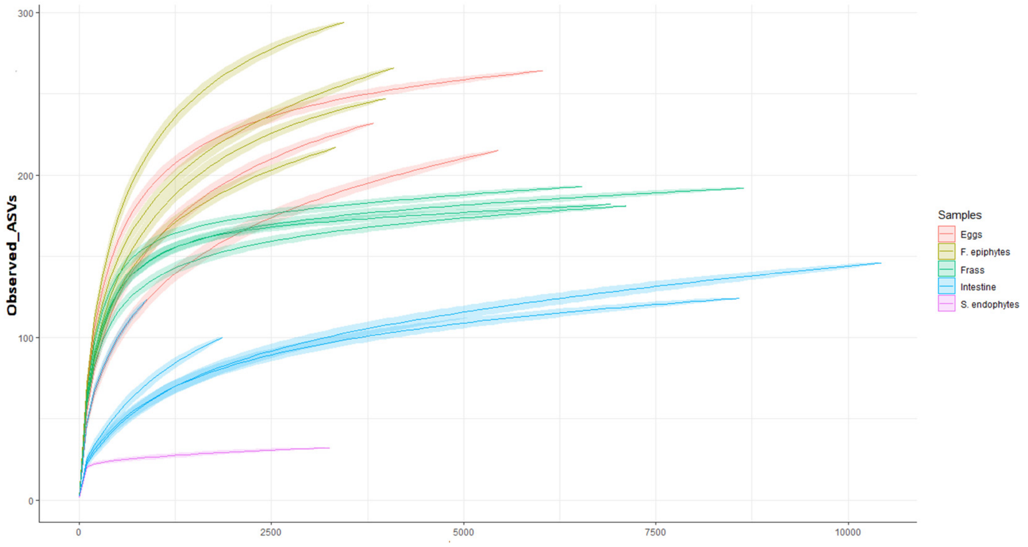

Figure S1. Rarefaction curves of the observed ASVs in the eleven samples of the insect and the five samples of the plant. Due to the few identified ASVs, foliar endophyte samples were discarded from the analysis of diversity and only one seed sample was kept in it.

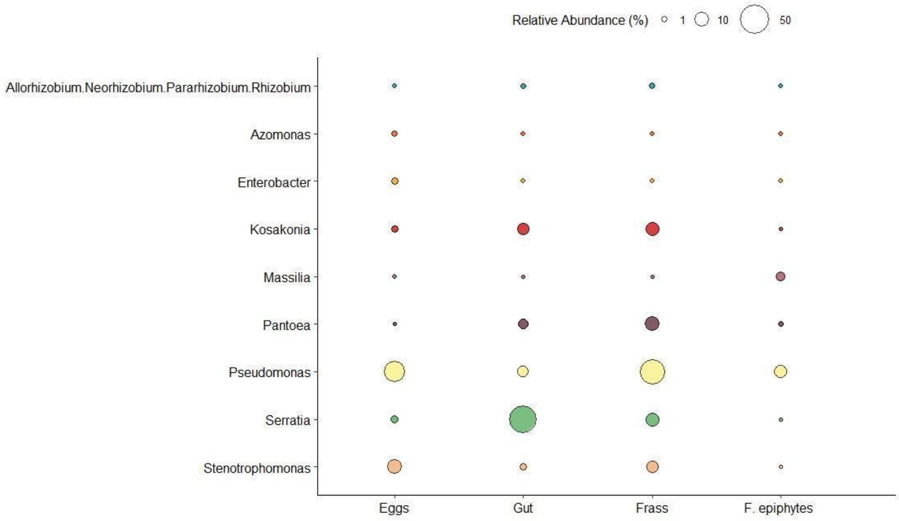

Figure S2. Relative abundance of some genera shared among insects samples (eggs, guts and frass) and plant samples (foliar endophytes). Only shared ASVs with abundances greater than 0.1% are shown.

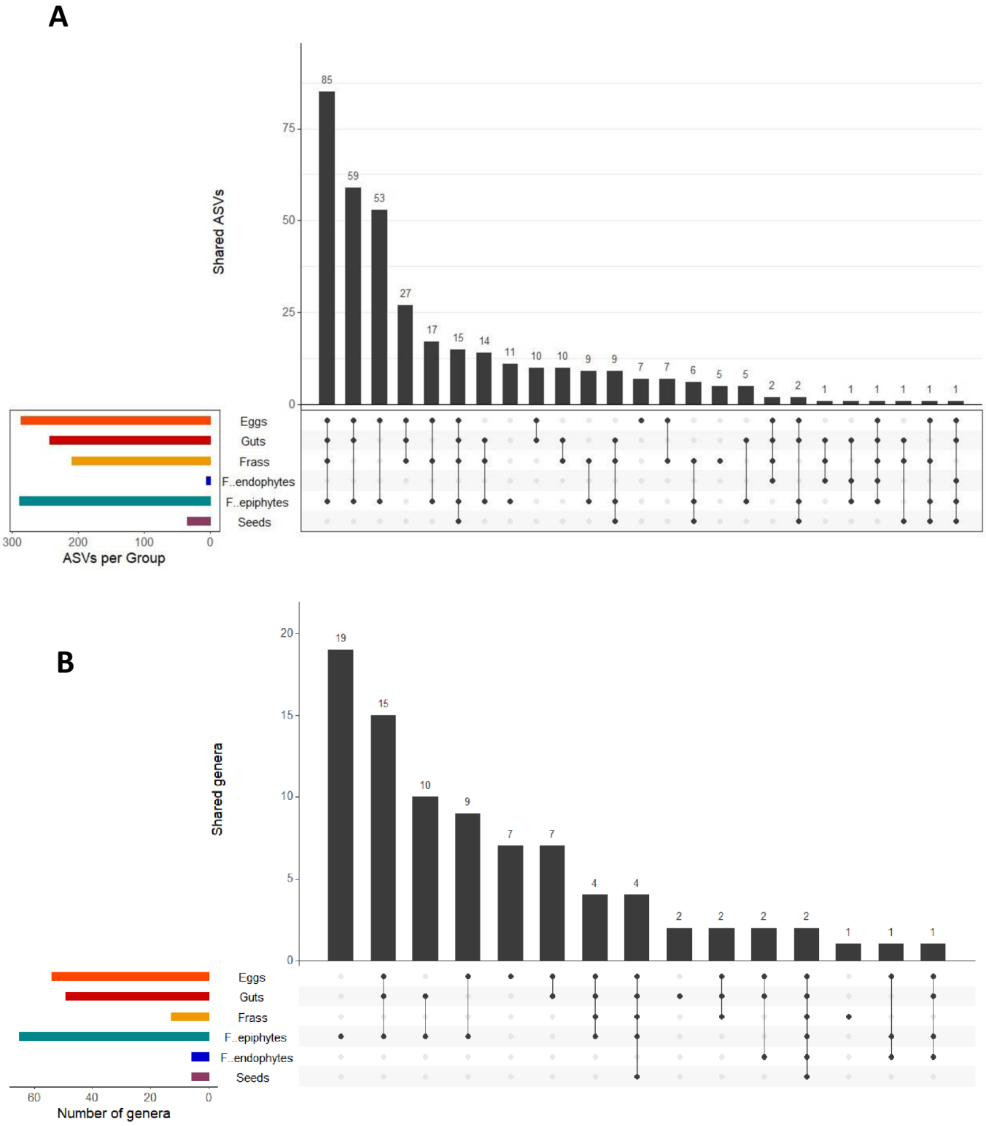

Figure S3. Shared ASVs (A) and genera (B) between insect and plant samples. Only shared ASVs and genera with abundances greater than 0.1% are shown.

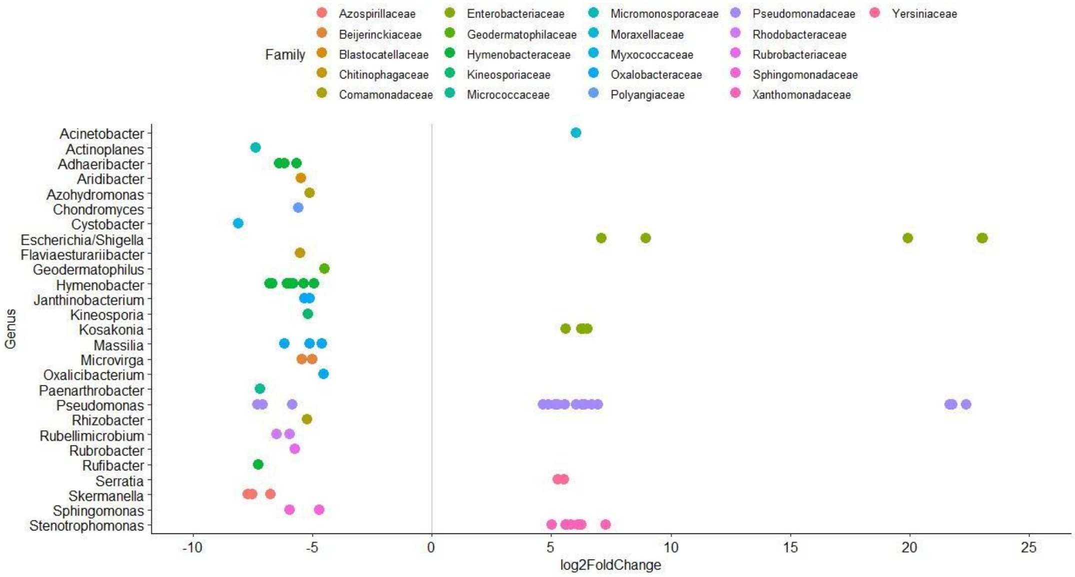

Figure S4. Genera with significant differences in abundances when comparing insect (eggs, gut and frass) and plant (foliar epiphytes) samples. The x-axis represents the values of log2FoldChange. Negative values indicate that the abundances of such genera are higher in the plant. Positive values indicate those genera are more abundant in the insect. This comparison was made with the DESeq2 function, with a Wald test and a $P < 0.01$.

Supplementary Tables

Table S1. Summary of the sequences and ASVs analyzed per sample.

sample	input	filtered	denoisedF	nonchim	# reads	# ASV
seeds1	37483	12836	12801	11622	5	5
seeds2	36103	12748	12680	11235	3262	32
seeds3	40324	15056	14981	13749	0	0
seeds4	45385	14652	14617	13264	1	1
f_epiphytes1	40592	12671	12215	10849	4098	266
f_epiphytes2	37271	12128	11466	10688	3345	217
f_epiphytes3	33386	11242	10857	9908	3985	247
f_epiphytes4	31849	8718	8041	7473	3452	294
f_endophytes1	32208	13081	13071	12247	2	2
f_endophytes2	35438	12349	12332	11558	2	2
f_endophytes3	40358	13869	13857	12954	0	0
f_endophytes4	31146	11292	11284	10679	3	3
eggs1	35702	12749	12408	11100	6035	264
eggs2	29761	9033	8929	7976	5450	215
eggs4	27033	10246	10141	9211	3828	232
gut1	41745	14095	14011	13491	1870	100
gut2	45994	16286	16204	15795	873	123
gut3	46433	15735	15669	14647	8589	124
gut4	37873	13081	13038	11669	10431	146
frass1	34608	11589	11501	9540	8644	192
frass2	27645	9529	9441	7894	7107	181
frass3	27644	9625	9545	8051	6543	193
frass4	31103	9698	9632	8613	6919	182

Table S2. Shared genera among the plant bacteriome with different combinations of the insect samples. Only shared ASVs with abundances greater than 0.1% are shown.

Sample combinations	# ASVs	# Genera	Genera
all plant samples & eggs	257	38	*Allorhizobium-Neorhizobium-Pararhizobium-Rhizobium, Aureimonas, Azomonas, Bacillus, Blastococcus, Brucella, Clostridium, Delftia, Enterobacter, Escherichia/Shigella, Gaiella, Hymenobacter, Janthinobacterium, Kineococcus, Kosakonia, Lawsonella, Lechevalieria, Leifsonia, Marmoricola, Massilia, Methylobacterium-Methylorubrum, Microbacterium, Microvirga, Modestobacter, Nocardioides, Oxalicibacterium, Pantoea, Paraclostridium, Paracoccus, Pseudomonas, Pseudocardia, Roseomonas, Serratia, Siccibacter, Sphingobacterium, Sphingomonas, Stenotrophomonas, Streptococcus*
all plant samples & guts	197	37	*Allorhizobium-Neorhizobium-Pararhizobium-Rhizobium, Azomonas, Bacillus, Brucella, Enterobacter, Escherichia/Shigella, Geodermatophilus, Hymenobacter, Janthinobacterium, Kineococcus, Kocuria, Kosakonia, Lawsonella, Lechevalieria, Leptolyngbya, Marmoricola, Massilia, Methylobacterium-Methylorubrum, Microbacterium, Microvirga, Modestobacter, Nocardioides, Noviherbaspirillum, Paenarthrobacter, Pantoea, Paraclostridium, Paracoccus, Parviterribacter, Pseudomonas, Rickettsiella, Roseomonas, Rubellimicrobium, Serratia, Siccibacter, Skermanella, Sphingomonas, Stenotrophomonas*
all plant samples & frass	188	10	*Allorhizobium-Neorhizobium-Pararhizobium-Rhizobium, Azomonas, Enterobacter, Kosakonia, Massilia, Pantoea, Pseudomonas, Serratia, Siccibacter, Stenotrophomonas*
foliar epiphytes & all insect samples	85	15	*Allorhizobium-Neorhizobium-Pararhizobium-Rhizobium, Azomonas, Enterobacter, Kosakonia, Massilia, Pantoea, Pseudomonas, Serratia, Siccibacte, Stenotrophomonas*
